# Breast cancer cell line MCF7 escapes from G1/S arrest induced by proteasome inhibition through a GSK-3β dependent mechanism

**DOI:** 10.1038/srep10027

**Published:** 2015-05-05

**Authors:** Elena Gavilán, Servando Giráldez, Inmaculada Sánchez-Aguayo, Francisco Romero, Diego Ruano, Paula Daza

**Affiliations:** 1 Instituto de Biomedicina de Sevilla (IBIS)-Hospital Universitario Virgen del Rocío, Universidad de Sevilla, Sevilla, Spain; 2 Departamento de Bioquímica y Biología Molecular, Facultad de Farmacia, Universidad de Sevilla, Sevilla, Spain; 3 Departamento de Microbiología, Facultad de Biología, Universidad de Sevilla, Avenida Reina Mercedes 6, Sevilla, Spain; 4 Departamento de Biología Celular, Facultad de Biología, Universidad de Sevilla, Avenida Reina Mercedes 6, Sevilla, Spain

## Abstract

Targeting the ubiquitin proteasome pathway has emerged as a rational approach in the treatment of human cancers. Autophagy has been described as a cytoprotective mechanism to increase tumor cell survival under stress conditions. Here, we have focused on the role of proteasome inhibition in cell cycle progression and the role of autophagy in the proliferation recovery. The study was performed in the breast cancer cell line MCF7 compared to the normal mammary cell line MCF10A. We found that the proteasome inhibitor MG132 induced G1/S arrest in MCF10A, but G2/M arrest in MCF7 cells. The effect of MG132 on MCF7 was reproduced on MCF10A cells in the presence of the glycogen synthase kinase 3β (GSK-3β) inhibitor VII. Similarly, MCF7 cells overexpressing constitutively active GSK-3β behaved like MCF10A cells. On the other hand, MCF10A cells remained arrested after MG132 removal while MCF7 recovered the proliferative capacity. Importantly, this recovery was abolished in the presence of the autophagy inhibitor 3-methyladenine (3-MA). Thus, our results support the relevance of GSK-3β and autophagy as two targets for controlling cell cycle progression and proliferative capacity in MCF7, highlighting the co-treatment of breast cancer cells with 3-MA to synergize the effect of the proteasome inhibition.

Cancer development is often due to perturbations in the cell cycle that lead to unlimited proliferation and cancer cells are usually chemo-resistant^1^,^2^,^3^. Understanding how cells die is critical to develop new strategies in order to try to improve the therapies to kill tumor cells.

The ubiquitin-proteasome pathway is responsible for the degradation of most poly-ubiquitinated proteins including proteins that control cell cycle progression, death cell and in general all the proteins that confer normal homeostasis levels. Therefore, targeting the ubiquitin-proteasome pathway has emerged as a rational approach in the treatment of human cancers in the last years^4^,^5^,^6^. Moreover, because cancer cells are generally more sensitive than normal cells to the inhibition of proteasome activity^7^,^8^,^9^, proteasome inhibitors are being used in anti-cancer therapy.

On the other hand, autophagy constitutes one of the major responses of cells to external or internal stimuli. Autophagy is a cellular process that engulfs organelles and cytoplasmic contents to digest and recycle these materials to sustain cellular metabolism^10^,^11^,^12^. In addition to provide a basic catabolic function, autophagy is also used by the cell to cope with stressful conditions to improve survival^13^. As any other major phenomenon of cell biology, autophagy can be perturbed in cancer cells and it is also modulated by anticancer chemo-therapies^14^,^15^. In this sense, the role of autophagy is controversial and it seems to be both tumor cell line-and treatment-dependent. The link between autophagy and cell death is still ambiguous, and autophagy may serve as a tumor suppressor mechanism, directing the cells to self-destruction, or as an oncogenic process and hence avoiding cell death^14^,^15^,^16^,^17^,^18^. Remarkably, autophagosomal markers are overexpressed in breast carcinomas with different cytosolic patterns and prognosis^19^. Thus, a better comprehension of the role of autophagy in cancer cells is mandatory for chemo-therapy development. In addition, glycogen synthase kinase-3 beta (GSK-3β) is a serine/threonine kinase that has been extensively studied because of its roles in several physiological disorders including cancer^20^,^21^,^22^ and many data support a function for this protein as a cell cycle-key regulator^23^.

Here we have focused on both the effect of proteasome inhibition on cell cycle progression, investigating the role of GSK-3β, as well as the role of autophagy on cell proliferation under proteasome stress. We demonstrated that GSK-3β signaling is involved in G2/M arrest in MCF7 cell line under proteasome stress and identified autophagy as a cellular mechanism to evade cell cycle arrest in these cells. The lethal effect of MG132 on MCF7 cells is remarkably boosted by the inhibition of autophagy. Present findings support that blockade of autophagy may enhance the therapeutic effects of proteasome inactivation in the treatment of breast cancer.

## Results

### Proteasome inhibition arrested the cell cycle at G1 or G2/M phases in MCF10A and MCF7, respectively

**Figure 1:**
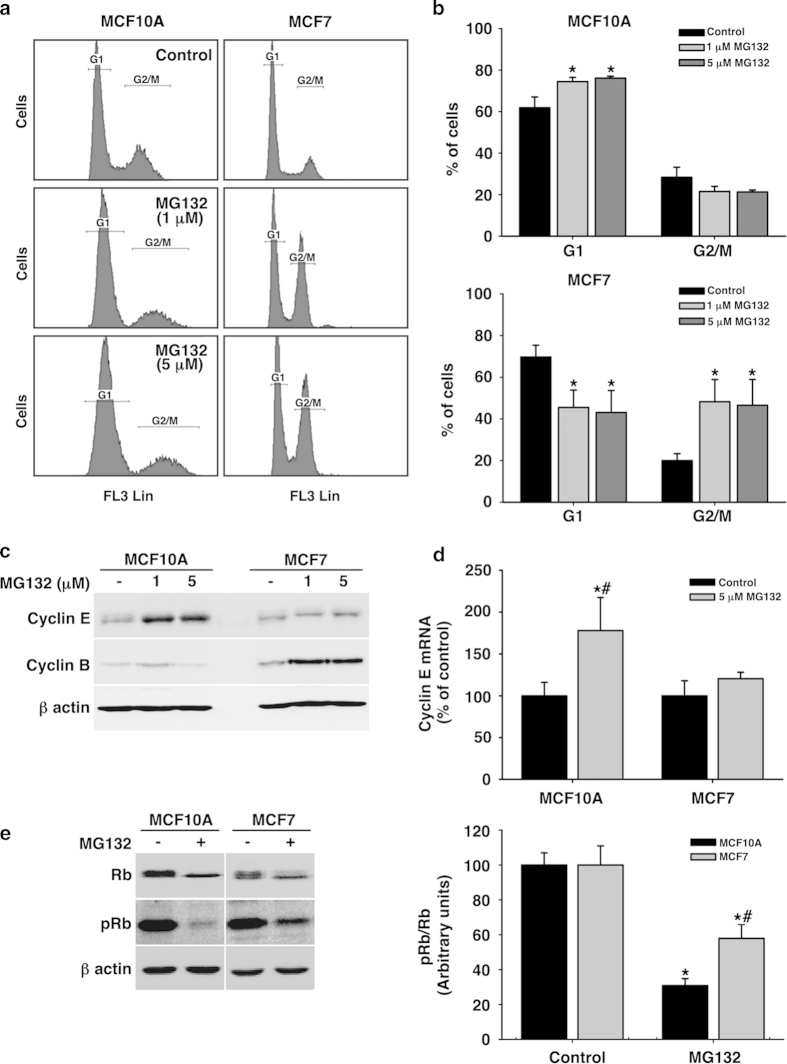
**Effect of proteasome inhibition on cell cycle in MCF10A and MCF7 cells. a**. Cell cycle distribution of both cell lines following 24 hours of MG132 treatment (1 and 5 μM). **b**. Quantification of cell cycle distribution indicating the % of cells detected in each stage (G1 and G2/M). Data are expressed as a percentage ± SD of three independent experiments. **c**. Representative western blots corresponding to the expression of cyclins E and B following MG132 (1 and 5 μM) for 24 hours. **d**. RT Real-time-PCR of cyclin E in treated and non-treated cells with 5 μM of MG132 for 24 hours. Data are expressed as a percentage ± SD of three independent experiments. **e**. Representative western blots of total and phospho-(Ser795) Rb in basal and 24 hours after 5 μM MG132 in both cell lines. It is shown also the quantification of the pRb/Rb ratio. Data are expressed as relative units of the optical density (OD) ± SD of three independent experiments. *significant differences compared to controls. # significant differences between MCF10A and MCF7 cells. Gels were run in parallel and under the same experimental conditions.

To evaluate the effect of the proteasome inhibitor MG132 on the cell cycle we treated both MCF10A, a normal mammary cell line, and MCF7, a breast tumor cell line, with MG132 1 and 5 μM for 24 hours and afterwards, cells were analyzed by flow cytometry. As shown in Fig. 1a, it can be noted that while in MCF10A cells both doses caused a significant arrest in G1 (P = 0.002), the tumor cell line MCF7 seemed to overtake the G1 checkpoint and were mainly arrested in the G2/M phase (Fig. 1a,b).

To further confirm these data we analyzed by western blots the expression of two specific cell cycle proteins: cyclin E (G1/S) and cyclin B (G2/M). Proteasome inhibition induced cyclin E accumulation, in normal but not in tumor cell line. By contrast, cyclin B accumulated in tumor cells, but not in the normal mammary epithelial cells, supporting flow cytometry data (Fig. 1c). Remarkably, proteasome inhibition induced significant transcriptional expression of cyclin E in MCF10A but not in MCF7 cells (P = 0.002; Fig. 1d). We further analyzed the level of phosphorylation of the Rb protein. As shown in Fig. 1e, the amount of both Rb and phospho-Rb was similar in both cell types under control conditions. However, MG132 treatment produced a significant decrease in the Rb/phospho-Rb ratio in normal mammary versus tumor cells (P = 0.02). Taken together, these results show that under proteasome stress the tumor cell line MCF7 is overriding the G1 checkpoint and progressing through the S phase to finally arrest in the G2/M phase.

### GSK-3β inhibition rescued MCF10A cells from G1/S arrest induced by proteasome inhibition

**Figure 2:**
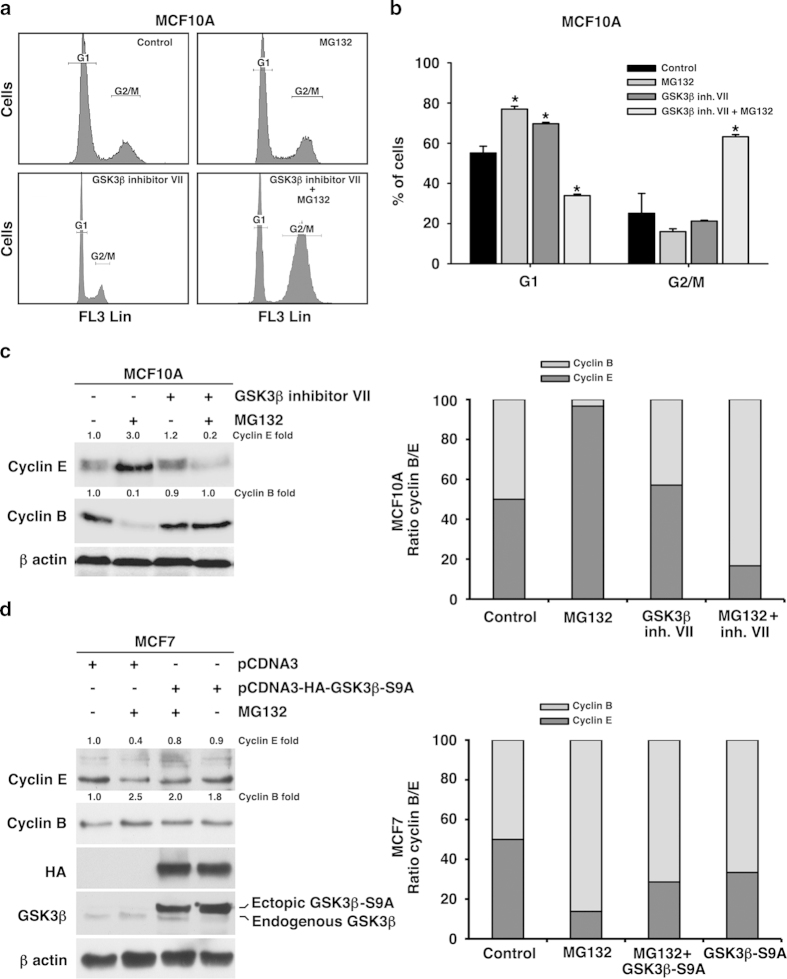
**Cell cycle behavior of both cell lines after GSK-3** β **modulation. a**. Cell cycle distribution of MCF10A cells following 24 hours of 1 μM MG132 and 20 μM GSK-3β inhibitor VII independently or combined. **b**. Quantification of the cycle distribution indicating the % of cells detected in each stage (G1 and G2/M). Data are expressed as a percentage ± SD of three independent experiments. **c**. Representative western blots corresponding to the expression of cyclins E and B in MCF10A cells in the presence of both MG132 and GSK-3β inhibitor VII, independently or combined, and a diagram of the cyclin B/E ratio. **d**. Representative western blot of cyclins E and B in GSK-3β -S9A-transfected MCF7 cells treated with 1 μM MG132, and a diagram of the cyclin B/E ratio. The quantitative fold change in cyclin E or B was determined relative to the loading control. *significant differences compared to controls. Gels were run in parallel and under the same experimental conditions.

We have previously shown that acute proteasome inhibition produced a consistent and dose dependent inhibition of the GSK-3β protein in the tumor cell line MCF7 but not in the normal mammary cell line MCF10A^24^. Because growing evidence indicated that GSK-3β can be, directly or indirectly, involved in cell cycle progression we wondered whether cell cycle behavior of tumor cells under proteasome stress could be dependent, at least in part, on GSK-3β signaling. To address this issue, we treated the cell line MCF10A with 1 μM of MG132 for 24 hours, in the absence or in the presence of 20 μM of the GSK-3β inhibitor VII. As show in Fig. 2a and B, GSK-3β inhibitor VII overtook the G1/S arrest of MCF10A cells treated with MG132, and detained cell cycle at G2/M phase (P = 0.001), similarly as tumor cells did in the presence of MG132 alone (see Fig. 1a). Moreover, western blot analysis revealed that expression of cyclins E and B in the presence of both MG132 and GSK-3β inhibitor VII (Fig. 2c) was similar to that observed in MCF7 cells in the presence of MG132 alone (see Fig. 1c). In this sense, the cyclin E/cyclin B ratio was high in MCF10A cells treated with MG132 but low in co-treated MCF10A cells, similarly to MG132 treated MCF7 cells (Fig. 2c right). To go further, we transfected MCF7 cells with pCDNA3-HA-GSK-3β-S9A, that codifies a constitutively active form of GSK-3β, in order to enhance the level of active GSK-3β protein in the tumor cells. As it is shown in Fig. 2d, western blot analysis revealed that decreased expression of cyclin E induced by proteasome inhibition in MCF7 cells was abolished in pCDNA3-HA-GSK-3β-S9A transfected cells. Similarly, increased cyclin B expression was partially reverted in MCF7 transfected cells, as well as the G2/M arrest (Fig. 2d right and Supplementary Fig S1). Thus, these data strongly support that differences in the cell cycle behavior between both cell lines, induced by proteasome inhibition, are mediated at least in part, by GSK-3β signaling.

### Tumor cell line MCF7 recovered the proliferative capability after removing MG132 while MCF10A cells remained arrested

**Figure 3:**
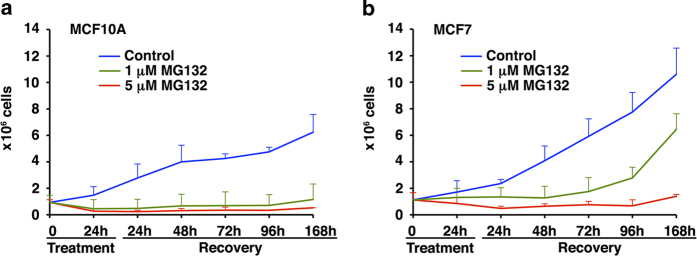
**Analysis of cell recovery following proteasome inhibition.** It is shown the proliferative capacity of MCF10A (**a**) and MCF7 (**b**) cells during a week after removing MG132 (1 and 5 μM). Data are expressed as average ± SD of cell number. Note MCF7 recovery following 1 μM of MG132, while this is not observed in MCF10A cells.

Based on the different behavior induced by proteasome inhibition in both cell types, we wondered whether molecular and cellular differences could affect the proliferative capability. To address this issue, we analyzed for a week the growing rate of both cell types after removing MG132 (1 and 5 μM). As shown in Fig. 3a,b, control cells were growing properly, while MG132 treatment drastically affected cell proliferation in both cell lines. The higher dose of MG132 caused similar effects on both cell types and neither proliferation nor recovery was observed after a week. However, cellular response to the lower dose of MG132 was quite different in both cell types. While in MCF10A cells, proliferation was very limited (around 10% of control cell number at the end of the assay), the tumor cells started to proliferate 3 to 5 days after MG132 removal, reaching up to the 60% of control cell number at the end of the assay (Fig. 3a,b, respectively). These results indicated that tumor cells escaped from the cell cycle arrest induced by MG132 treatment, while MCF10A did not.

### Proteasome stress induced early autophagy activation in MCF7 cells

**Figure 4:**
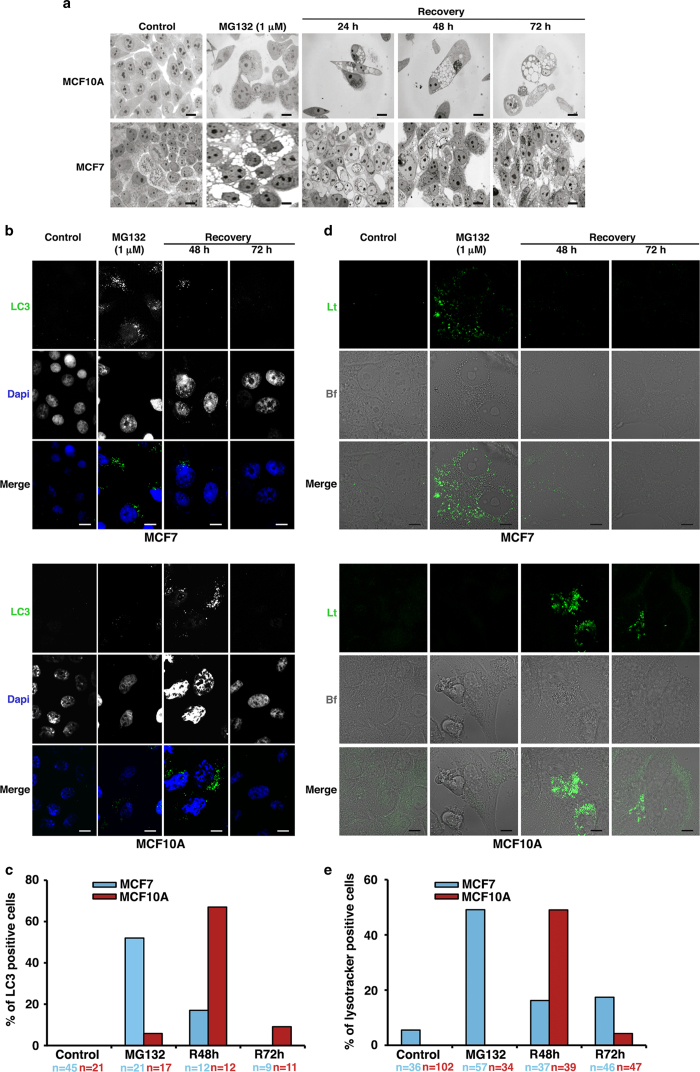
**Study of the proteasome inhibition and recovery in MCF7 and MCF10A cells. a**. Structural study of both cell lines 24, 48 and 72 hours after MG132 withdrawal. Note the high vacuolization level in MCF10A cells. Scale bar 10 μm. **b**. Control MCF7 and MCF10A cells, treated with MG132 1 μM for 24 hours and recovered 48 and 72 hours later were stained for LC3 and DNA. In the merge, LC3 staining is shown in green and DNA in blue. Scale bar 10 μm. **c.** Quantification of LC3 positive cells from B. Cells were randomly selected from the acquired image and counted. The cells with more than five dots of specific green signal were considered to be LC3 positive. n = number of cells analyzed. **d**. Confocal microscopy of LysoTracker Green localization in MCF7 and MCF10A cells treated as in B. Lt: LysoTracker, Bf: Bright field. Scale bar 5 μm. **e**. Quantification of LysoTracker positive cells from D. Cells were randomly selected from the acquired image and counted. n = number of cells analyzed. R48h and R72h represent data from 48 and 72 hours after MG132 release.

Since cell recovery after MG132 treatment was different in both cell lines, we performed morphological, ultrastructural and biochemical analysis after removing MG132. The study was carried out just along three days after MG132 withdrawal. As expected 24, proteasome inhibition (MG132 1 μM for 24 hours) induced the formation of cytosolic aggregates in MCF10A cells, while induced cytosolic vacuoles in MCF7 cells (Fig. 4a). In order to test whether these cytosolic vacuoles corresponded to autophagosomes, we performed immunofluorescence assay with the autophagosomal marker LC3. As shown (Fig. 4b upper panel), proteasome inhibition induced early formation of perinuclear punctated structures immunopositive for LC3 in tumor cells. The presence of these structures diminished in a time dependent manner during the recovery period. By contrast, in MCF10A cells, similar punctated structures were observed but they appeared during the recovery period (Fig. 4b lower panel). Quantification of LC3 immunopositive cells in both MCF10A and MCF7 lines is shown in Fig. 4c. These data suggest that autophagy activation induced by proteasome inhibition started earlier in MCF7 than in MCF10A cells. This idea was corroborated by the staining assay with the acidotropic dye LysoTracker that labels acidic compartments such as lysosomes and autophagosomes. The staining pattern was similar to that observed for LC3 in both cell types (Fig. 4d,e).

**Figure 5:**
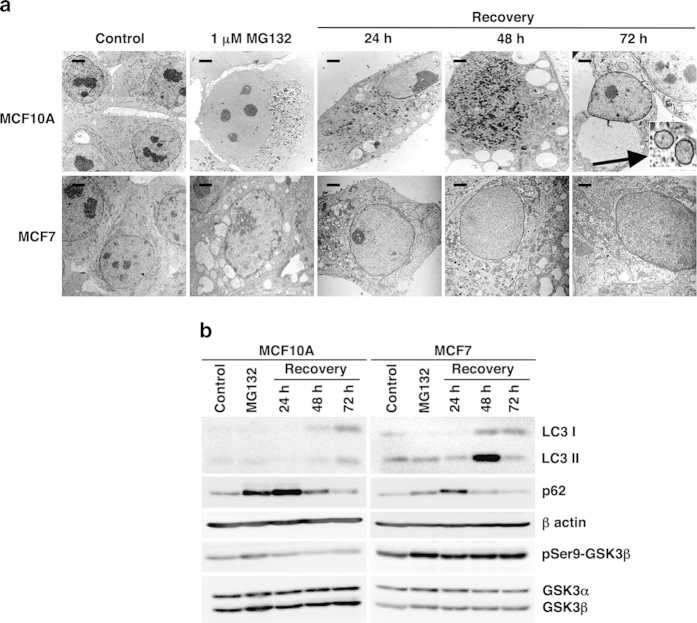
**Ultrastructural and biochemical analysis of autophagy resolution during proteasome inhibition recovery. a**. TEM analysis of MCF10A (upper panel) and MCF7 cells (lower panel) following 24 hours of proteasome inhibition and 24, 48 and 72 hours after removing MG132. Scale bar 4 μm. **b**. Representative western blots of specific autophagic markers (LC3 and p62) and phosphorilated GSK-3β protein expression in MCF10A (left) and MCF7 cells (right), following 24 hours of proteasome inhibition and 24, 48 and 72 hours after removing MG132. Gels were run in parallel and under the same experimental conditions.

On the other hand, MCF10A cells augmented in size and diminished in number after MG132 elimination. Moreover, the yuxtanuclear aggregates were disappearing at the same time that cytosolic vacuoles were appearing throughout the recovery period, showing most of them a highly vacuolated cytoplasm. This was also confirmed by ultrastructural analysis by TEM in ultrathin sections. As shown in Fig. 5a, cytoplasmic vacuoles in MCF10A cells increased in size, in a time dependent manner, filling the entire cytosolic surface and surrounding the cytoplasmic aggregates in some cells. Some cytosolic vacuoles observed in MCF10A cells had the appearance of autophagic vacuoles as judged by the double membrane and the heterogeneous content, supporting immunofluorescence data. In addition, we also observed large empty vacuoles in both cell types which resembled senescent vacuoles. In fact, the proportion of senescent cells, determined by SA-β-gal assay, was significantly higher in MCF10 than in MCF7 cells 48 hours following MG132 removal (P = 0.0003; Supplementary Fig S2).

To better characterize the autophagy activity, we performed western blot analysis of both LC3 II and p62 proteins. In MCF10A cells, MG132 did not modify the amount of LC3 II, but increased the content of p62 even 24 hours after MG132 elimination, supporting absence of autophagy activation (Fig. 5b). However, during the recovery period LC3 II increased at the same time that p62 decreased, supporting late autophagy activation. In contrast, in MCF7 cells, MG132 increased basal autophagic activity with minor p62 accumulation. Remarkably, a dramatic LC3 II accumulation occurred 48 hours following MG132 removal, which returned to basal level 24 hours later (Fig. 5b). This might be due to a decrease in the autophagy flux in MCF7 (Supplementary Fig S3). However, we cannot rule out other possibilities. Importantly, tumor cells showed a long-term GSK-3β inhibition during the recovery phase that was not observed in the MCF10A line. Taken together, these results strongly support that early autophagy activation in addition to GSK-3β chronic inhibition might be crucial for tumor cell recovery under proteasome stress.

### Recovery of the proliferative capacity was dependent on early autophagy activation

**Figure 6:**
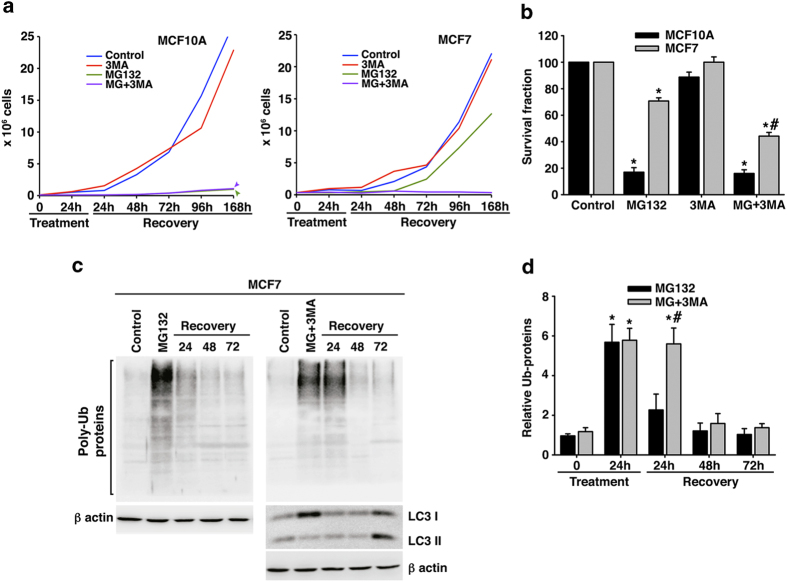
**Comparative study of the effect of the co-treatment with proteasome and autophagy inhibitors. a**. Proliferative capacity of MCF10A (left) and MCF7 cells (right) during a week following 24 hours of MG132 and 3-MA treatment, independently or combined. Note that MCF7 cells do not recover the proliferative capacity with the co-treatment, compared with MCF7 cells treated only with MG132. **b**. Clonogenic assay of MCF10A and MCF7 cells for fifteen days after MG132 and 3-MA withdrawal, independently or combine. Data were similar to those obtained with the growth curves. **c**. Representative western blots of total poly-ubiquitinated proteins in MCF7 cells 24, 48 and 72 hours after removing MG132 (left) or after co-treatment removal (right). It is also shown a representative western blot of LC3 protein expression during co-treatment recovery. **d**. Quantification of total poly-ubiquitinated proteins in MCF7 cells as shown in **c**. Data are expressed as average ± SD of relative units of three independent experiments. *significant differences compared to control cells. # significant differences between MG132-treated and co-treated cells. Gels were run in parallel and under the same experimental conditions.

To evaluate the role of early autophagy activation in the recovery of the proliferative capacity of cells under proteasome stress, we treated MCF7 and MCF10A cells with MG132, 3-MA or both for 24 hours and, after elimination of the corresponding compounds, we performed proliferation assays. As shown in Fig. 6a, the treatment with the autophagy inhibitor 3-MA did not affect the cell proliferation rate neither in MCF10A nor in MCF7 cells. On the other hand, MG132 strongly affected cell proliferation in MCF10A whereas MCF7 cells were able to proliferate reaching up to 57% of control cell number. However, co-treatment of MG132 and 3-MA abolished cell proliferation in the tumor cell line, but growth rate was not modified in the normal mammary cell line (compared to MG132 treatment). In order to confirm this issue we performed clonogenic assays for fifteen days using the same experimental conditions. As it is shown in Fig. 6b, the proliferative capability of both cell lines was affected by proteasome inhibition, being the normal mammary cells the most altered. Nevertheless, the number of MCF7 cell colonies diminished significantly (P = 0.0001) 15 days after co-treatment of MG132 and 3-MA (around 40% compared to MG132 treated MCF7 cells), while in the MCF10A cells the survival fraction remained constant, suggesting that early autophagy activation is relevant for the recovery of cell proliferation in the tumor cell line MCF7, while late autophagy activation is not so profitable for MCF10A cells. We further analyzed at the molecular level the effect of MG132 and 3-MA co-treatment in the tumor cell line MCF7. As shown in Fig. 6c, both proteasome and autophagy inhibition induced a significant increase in the content of poly-ubiquitinated proteins (P = 0.0001). Moreover, co-treatment modified the profile of poly-ubiquitinated protein accumulation, indicating that 3-MA was efficiently inhibiting autophagy in these cells. The clearance of poly-ubiquitinated proteins was significantly delayed in co-treated MCF7 cells, compared to cells treated with MG132 alone (P = 0.0002; Fig. 6d). This difference could be attributable to the early block of autophagy produced by 3-MA. Indeed, at 24 hours of co-treatment we observed the accumulation of LC3 I but not of LC3 II, supporting autophagy inhibition. Importantly, autophagy activity was restored in co-treated MCF7 cells during the recovery phase as judge by the level of LC3 II, which was observed 24 hours later than in MCF7 cells treated with MG132 alone (compare Fig. 5b upper panel with Fig. 6c lower panel). Taken together, these findings strongly support that basal autophagy activity in the breast cancer cell line MCF7 represents a mechanism to evade cell death induced by proteasome inhibition.

### GSK-3β inhibition partially recovered the proliferative capacity of MCF10A cells under proteasome stress

**Figure 7:**
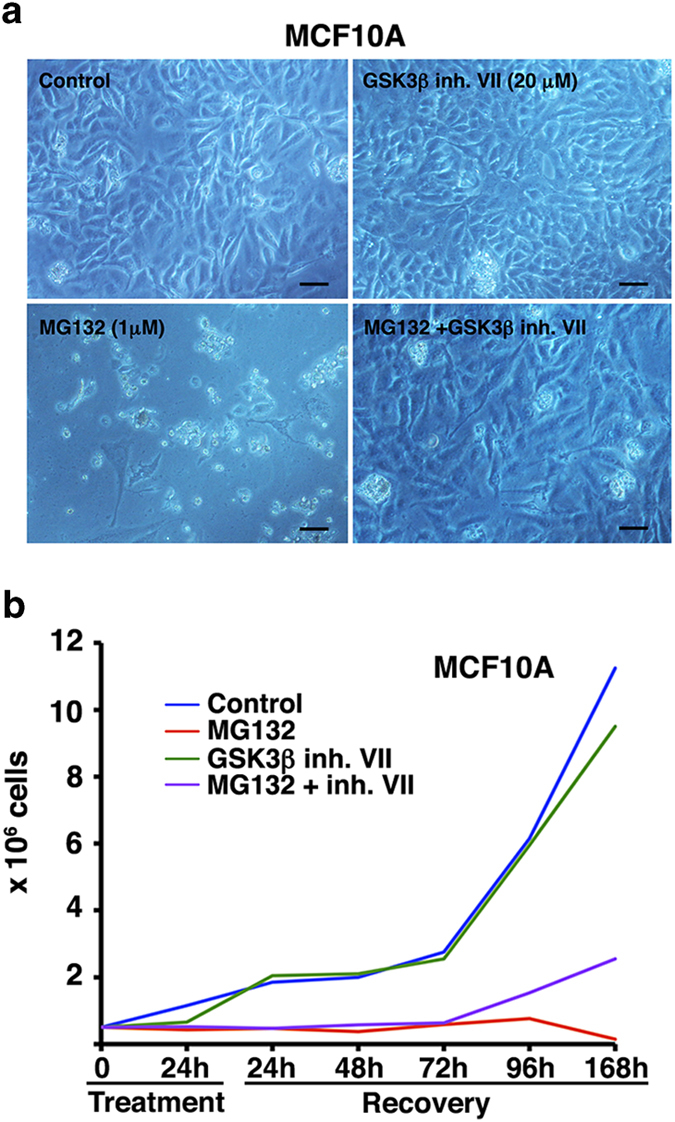
**Analysis of MCF10A cell recovery following proteasome inhibition with MG132 and in the presence of the GSK-3**β **inhibitor VII. a.** Representative images of MCF10A cells in control situation and with different treatments at the end of the assay. Note the difference in cell number in MG132 (24 h) and MG132 (24 h) + GSK-3β inhibitor VII (present during treatment and recovery). Scale bar 30 μm **b**. Proliferative capacity of MCF10A cells during a week following 24 hours of MG132 1 μM and GSK-3β inhibitor VII 20 μM treatment, independently or combined. Note that cells treated with MG132 do not recover the proliferative capacity while co-treated cells (MG132 + GSK-3β inhibitor VII) restored cell proliferation rate.

As showed above, GSK-3β inhibition rescued MCF10A cells from G1/S arrest induced by MG132 (see Fig. 2). Importantly, as we and other have previously demonstrated pharmacological inhibition of GSK-3β activity induced early autophagy activation^24^,^25^. In order to test whether this inhibition in MCF10A cells would mimic MCF7 proliferative behavior following 24 hours of MG132 treatment, we inhibit GSK-3β and performed proliferation assays. Treatment with the GSK-3β inhibitor for 168 hours did not affect cell proliferation rate, while proteasome inhibition deeply impaired cell growth even 168 hours after drug removal (Fig. 7a). However, when MCF10A cells were simultaneously treated with both inhibitors for 24 hours, and cells were allowed to grow in the presence of the GSK-3β VII inhibitor after MG132 elimination, cell proliferation began to recover reaching up to 20% of control cell number 168 hours later (Fig. 7b). Thus, these data provide strong evidence supporting that chronic GSK-3β inhibition in addition to early autophagy activation could be responsible for the proliferation recovery of both breast epithelial cells following proteasome inhibition, a mechanism that could be essential for cell survival.

## Discussion

Breast cancer stands second in cancer-related mortality of women 26,27. In the present work we have investigated the role of proteasome inhibition on cell cycle progression and cell proliferation in both the human breast cancer cell line MCF7 and the normal mammary cell line MCF10A. The most relevant findings are summarized as follow: i) tumor cells ignored the G1/S but arrested in the G2/M checkpoint following proteasome inhibition, whereas the normal mammary cells arrested in the G1/S checkpoint; ii) GSK-3β inhibition was necessary to override the G1 checkpoint under proteasome stress; iii) tumor cells recovered the proliferative capability three days after MG132 was removed, while MCF10A did not; iv) tumor cells activated proteasome inhibition induced autophagy earlier than MCF10A; and v) early autophagy activity was necessary for the recovery of cell proliferation in the tumor cell line MCF7. Taken together our results support that GSK-3β signaling and autophagy activity are two related processes that confer adaptive advantages to cell survival.

Cyclins are the regulators of the cell cycle^28^,^29^,^30^,^31^. The cyclin E binds to the G1 phase cyclin-dependent kinase Cdk2 controlling the G1 to S phase transition in cell cycle, which is the rate-limiting step for proliferation^32^. Our results demonstrated that, under proteasome stress, cyclin E accumulated exclusively in MCF10A cells indicating arrest at the G1/S phase. Indeed, the pRb/Rb was significantly lower in these cells than in tumor cells, supporting that cyclin E/Cdk2 complex was not active in MCF10A. On the other hand, cyclin B levels raise during the S and G2 phases for entering M phase and subsequently cyclin B must be degraded by the proteasome in order to finish mitosis and enter G1 phase again^33^. In this sense, we demonstrated that proteasome inhibition caused accumulation of cyclin B only in MCF7 cells, supporting arrest at the G2/M phase. Moreover, the analysis of phospho-Rb revealed that in these cells remained always a significant amount of this phosphorylated protein, leading to progression through the G1 phase of the cell cycle^34^,^35^. These results point that the tumor cell line MCF7 fails to comply with G1 checkpoint, which was then overpassed under stressful conditions, allowing the cells to go further.

In addition, GSK-3β is also an important regulator of several processes, including cell cycle progression as well as proliferation and cell death^22^,^36^. In this sense, cyclin E levels are negatively regulated by GSK-3β, which phosphorylates it promoting its proteasomal degradation^37^,^38^. In fact, MCF10A cells have a high GSK-3β activity and proteasome inhibition increased the cyclin E/cyclin B ratio, arresting the cells in the G1/S phase. However, GSK-3β activity in MCF7 is lower than in MCF10A, and MG132 decreased the cyclin E/cyclin B ratio being the cells then arrested at G2/M. Remarkably, these features were causally related because of proteasome inhibition in MCF10A cells decreased the cyclin E/cyclin B ratio and arrested cells in the G2/M phase, as MCF7 cells did, when GSK-3β was pharmacologically inhibited. Similarly, proteasome inhibition in MCF7 cells that overexpressed a constitutive active form of GSK-3β, increased the cyclin E/cyclin B ratio as did MCF10A cells. Taken together these data indicated that under proteasome stress cyclin E degradation is depending on GSK-3β activity, suggesting that chronic inhibition of GSK-3β could be responsible, at least in part, of the cell cycle alteration in these tumor cells.

An important issue remaining is to know how tumor cells can progress when proteasome is inhibited. In this sense, proteasomal degradation of cyclin E is mandatory for cell cycle progression. Thus, additional pathways for the degradation of this protein must be operative in these tumor cells under proteasome stress. We speculate about the possibility that autophagy could be involved in this process. This speculation is based on the fact that MCF7, but not MCF10A cells, early increased the autophagy activity under proteasome stress, a process that was mediated by the inhibition of GSK-3β^24^,^25^. Here, we demonstrated that normal mammary cells behaved like tumor cells when GSK-3β was pharmacologically inhibited. In this way, under proteasome stress situation cyclin E accumulated in MCF10A because the proteasome was inhibited, whereas in the tumor cells MCF7 cyclin E did not accumulate because it might be being degraded by the autophagy pathway. It is noticeable, that cyclin B accumulated in MCF7 cells, indicating that proteasome was efficiently inhibited also in these cells. However, further experiments will be necessary to evaluate the potential role of autophagy in cyclins degradation.

Because cancer cells are generally more sensitive than normal cells to the inhibition of proteasome activity, proteasome inhibitors are being used in anti-cancer therapy. Furthermore, our results also demonstrated that cell proliferation was higher in tumor than in normal mammary cells. Importantly, we have previously demonstrated that acute proteasome inhibition produced a huge autophagy induction accompanied by a higher mortality rate in MCF7 than in MCF10A cells^24^. However, here we observed that tumor cells recovered better than MCF10A cells from proteasome inhibition. Indeed, the normal mammary cells did not proliferate at any doses used in the study, whereas MCF7 cells started to proliferate 3 days after removal of the lower dose of MG132. Our data also revealed that cell recovery was dependent on early autophagy activation and chronic GSK-3β inhibition. In fact, under proteasome stress tumor cells early activated autophagy, chronically inhibited GSK-3β and recovered cell proliferation, whereas MCF10A cells did not. However, when MCF10A cells were treated with MG132 in the presence of the GSK-3β inhibitor VII, they early activated autophagy^24^,^25^ and began to proliferate, resembling MCF7 behavior.

On the other hand, late autophagy activation induced by proteasome inhibition in normal mammary cells might be mediated by a GSK-3β-independent mechanism. In this sense, the unfolfed protein response might be an alternative^39^. This mechanism could be also operative in MCF7 cells. Importantly, induction of autophagy through ATF4 has been proposed as a relevant resistance mechanism to Bortezomib treatment in breast cancer cells^40^ and we have previously reported strong up-regulation of ATF4, and mostly CHOP, in MCF7 but not in MCF10A cells, 24 hours after proteasome inhibition^24^. Thus, we speculate that early activation of autophagy could be mostly dependent on GSK-3β inhibition and ATF4 expression, whereas late autophagy induction could be more dependent on the unfolded protein response.

In summary we provide solid evidence supporting that the simultaneous targeting of the proteasome and the autophagy machinery may represent an alternative for breast cancer treatment in order to promote a lethal synergic effect caused by the inhibition of both proteolytic systems.

## Methods

### Cells and culture conditions

The study was carried out in an ER + human epithelial breast cancer cell line, MCF7, and in a human epithelial normal mammary cell line, MCF10A (American Type Culture Collection). MCF7 cells were cultured in DMEM/Ham’s F12 (1/1) medium (PAA Laboratories) supplemented with 10% (v/v) foetal bovine serum (FBS) (Gibco), 2 mM L-glutamine, 50 μg/ml streptomycin and 50 U/ml penicillin (Sigma-Aldrich). The human normal mammary epithelial cells MCF10A were grown in DMEM/Ham’s F12 (1/1) medium (PAA Laboratories) supplemented with 10% (v/v) horse serum (Gibco), 2 mM L-glutamine, 50 μg/ml streptomycin, 50 U/ml penicillin, 2.5 mg/ml insulin, 150 μg/ml cholera enterotoxin (Sigma-Aldrich), 2.5 mg/ml hydrocortisone and 50 μg/ml epidermal growth factor (Calbiochem). Both cell lines were maintained at 37 °C in a humidified atmosphere with 5% CO2. Cells were routinely subcultured and they were always in exponential growth phase when used for experiments. Each experiment was independently performed at least in triplicate.

### Treatments and drugs

Both cell lines were treated in parallel with the reversible proteasome inhibitor MG132 (Sigma-Aldrich) at different doses. Cells were also treated with 1 mM of 3-MA (Sigma-Aldrich), an autophagy inhibitor and with 20 μM of GSK-3β inhibitor VII (Calbiochem). Treatments were carried out alone or in combination for 24 hours.

### Cell proliferation

#### Growth curves

Cells were plated in triplicate at a 60,000 cells/cm^2^ density in 35 mm dishes. Cells were allowed to adhere for two days and then they were treated with the different doses of MG132 (1 and 5 μM), 3-MA (1 mM) and GSK-3β inhibitor VII (20 μM) for 24 hours. After treatment, cells were washed twice with PBS and fresh medium was added to allow the cells to recover. When cells were treated with GSK-3β inhibitor VII, this was always present till the end of the assay to ensure a sustained inhibition of GSK-3β protein. The dishes were placed at the incubator for 168 hours; every 24 hours cells were trypsinized and counted with a haemocytometer. Cell viability was assessed by trypan blue exclusion test. Cells from dishes were counted by triplicate for each dose and in three independent experiments.

#### Clonogenic assays

Cells were trypsinized, counted and plated in triplicate in 12 mm dishes. Cell numbers seeded were 500 and 1,000 for controls and treatments respectively. Then, they were allowed to adhere for one day and they were treated with 1 μM MG132 and 1 mM 3-MA for 24 hours. After treatment, cells were washed twice with PBS and fresh medium was added. Cells were allowed to form colonies for 15 days. They were stained with methylene blue in 4% acetic acid for 30 min and washed with distilled water. Colonies with at least 50 cells were considered and scored using a colony counter.

### Western blotting

Immunoblots were done as previously described^41^. Briefly, proteins were loaded on a 10% or 12% polyacrylamide gel for electrophoresis (SDS–PAGE; Bio-Rad) and then transferred to a nitrocellulose membrane (Hybond-C Extra; Amersham). After blocking, membranes were incubated overnight at 4 °C, with the following primary antibodies: (i) rabbit polyclonal antibodies against: phospho-GSK-3β(S9) (Cell Signaling), Ubiquitin (Dako) , LC3 (Novus Biologicals) and p62 (Cell Signaling); (ii) mouse monoclonal antibodies against: β-actin (Sigma-Aldrich), GSK-3-clone 4G-1E (Millipore), cyclin E (HE12) and cyclin B1 (GNS1) from Santa Cruz Biotech., GSK-3β (BD Biosciences) and HA (anti-HA-peroxidase, Roche). Then, membranes were incubated with the appropriate secondary antibody (Dako) horseradish-peroxidase-conjugated, at a dilution of 1/10000 and developed using the ECL-plus detection method (Amersham) and the ImageQuant LAS 4000 MINI GOLD (GE Healthcare Life Sciences). For quantification, the optical density of individual bands was analyzed using PCBAS 2.08 software (Raytest Inc, Germany), and the optical density of each band was normalized relative to the optical density of β-actin.

### Morphological and ultrastructural studies

Cell monolayers were fixed in 2.5% (v/v) glutaraldehyde in 0.1 M sodium cacodylate-HCl buffer pH = 7.4 for 1h at 4 °C. Cells were rinsed in cacodylate buffer twice and incubated for 1h at 4 °C in 1% (v/v) OsO_4_/1% (w/v) K_4_Fe(CN)_6_ in cacodylate buffer pH = 7.4. Cells were rinsed again in cacodylate buffer and finally in distilled water. Then, cells were stained with 1% (w/v) aqueous uranyl acetate for 2h at 4 °C. After washing with distilled water, they were dehydrated through increased graded ethanol series and embedded in Epon resin 812. Ultrathin sections were stained with lead citrate for 5 min for transmission electron microscopy (TEM) (Philips CM10). Toluidine-blue stained 0.5 μm semithin sections were used as control for optical microscopy (Leica DME) and images were acquired 100X. All reagents were purchased from Sigma-Aldrich.

### RNA extraction and reverse transcription

For PCR analysis, total RNA was extracted using the Tripure Isolation Reagent (Roche, Mannheim, Germany), according to the instructions of the manufacturer. The recovery of RNA was similar between MCF10A and MCF7 cells. Reverse transcription was performed using random hexamers primers exactly as previously described^24^.

### Real-time RT-PCR

cDNAs were diluted in sterile water and used as template for the amplification by the polymerase chain reaction. Amplification of each specific gene product was performed using the ABI Prism 7000 sequence detector (Applied Biosystems) and TaqMan probes designed by Applied Biosystems. The cDNA levels of the different cell types were determined using two different housekeepers (GAPDH and β-actin). The amplification of the housekeepers was done in parallel with the gene to be analyzed. Similar results were obtained using both housekeepers. Thus, the results were normalized using both β-actin and GAPDH expression. Threshold cycle (Ct) values were calculated using the software supplied by Applied Biosystems.

### Immunofluorescence and LysoTracker assay

MCF7 and MCF10A cells were grown on coverslips, treated as indicated in the figure, fixed with 4% paraformaldehyde and permeabilized with 0.1% Triton X-100. Immunostaining, using a polyclonal anti-LC3 (Novus Biologicals) antibody, and counterstaining with DAPI to visualize the nuclei, was carried out according to standard protocols. Epifluorescence microscopy was performed using a Leica microscope. For LysoTracker assay, living cells, treated as indicated, were exposed to pre-warmed medium containing 50 nM of LysoTracker Green DND-26 for 30 min at 37 °C, washed with fresh medium and observed using a Zeiss confocal microscope.

### Flow cytometry analysis

MCF7 and MCF10A cells were treated with MG132 and GSK-3β inhibitor VII at the indicated doses for 24 hours. Cells were harvested by trypsinization and collected by centrifugation. Cells were washed once with PBS and fixed in 5 ml ethanol 70% at 4 ºC. Cells were washed once with PBS/1%BSA and then incubated with 1 ml of PBS/1%BSA containing 50 μg/ml propidium iodide and 0.1 mg/ml RNAse for 1h at room temperature. Cells were analyzed for DNA content by flow cytometry using a LSR Fortessa from Becton-Dickinson and doublet discrimination was performed. The data were analysed using the BD FACSDiva software.

### Transient transfection experiments

MCF7 cells were transiently transfected by electroporation with pCDNA3-HA GSK-3βS9A (Addgene) or empty vector (10 μg), incubated or not with MG132 (1 μM, Boston Chemical), and harvested 24 hours later.

### Statistical analysis

Data were expressed as mean ± SD. The comparison between both cell types was done by two-tailed t test. The significance was set at 95% of confidence. Statistical analysis was done using the Statgraphics plus (v 3.1) program (Warrenton, VA, USA).

## Author Contributions

P.D. and D.R. designed research and wrote the paper. E.G. performed main research and analyzed data. I.S-A. and P.D. performed morphological and ultrastructural studies. S.G. and F.R. performed the transient transfection experiments, the immunofluorescence and LysoTracker assays. All authors reviewed the manuscript.

## Additional Information

**How to cite this article**: Gavilán, E. *et al.* Breast cancer cell line MCF7 escapes from G1/S arrest induced by proteasome inhibition through a GSK-3β dependent mechanism. *Sci. Rep.* 5, 10027; doi: 10.1038/srep10027 (2015).

## Supplementary Material

Supplementary Figures
