# Supplementary material for: Breast cancer cell line MCF7 escapes from G1/S arrest induced by proteasome inhibition through a GSK-3β dependent mechanism
Source: Sci Rep. 2015 May 5;5:10027. doi: 10.1038/srep10027 (PMC4419540; doi:10.1038/srep10027)
Supplement: Supplementary Figures [file srep10027-s1.pdf]

Breast cancer cell line MCF7 escapes from G1/S arrest induced by proteasome inhibition through a GSK-3β dependent mechanism

Elena Gavilán, Servando Giráldez, Inmaculada Sánchez-Aguayo, Francisco Romero, Diego Ruano and Paula Daza

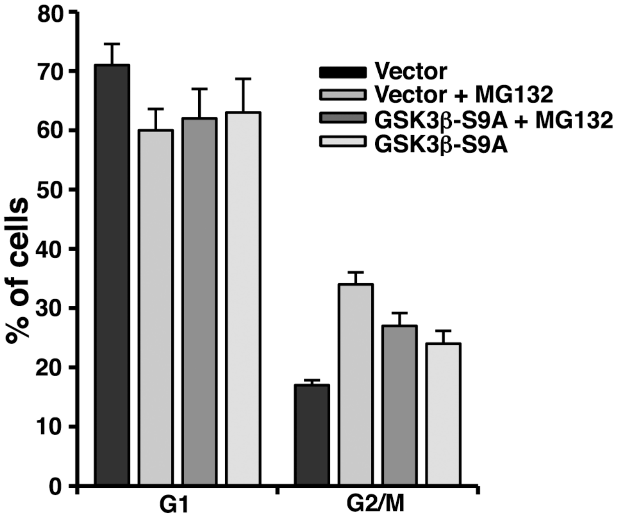

Supplementary Figure S1. Cell cycle analysis of proteasome inhibited GSK-3β-S9A-transfected cells. MCF7 cells transfected with pCDNA3-HA-GSK-3β-S9A or empty vector were treated or not with MG132 (1μM) for 24h and analyzed by flow cytometry. Quantification of cell cycle distribution indicating the % of cells detected in each stage (G1 and G2/M). Data are expressed as a percentage ± SD of three independent experiments.

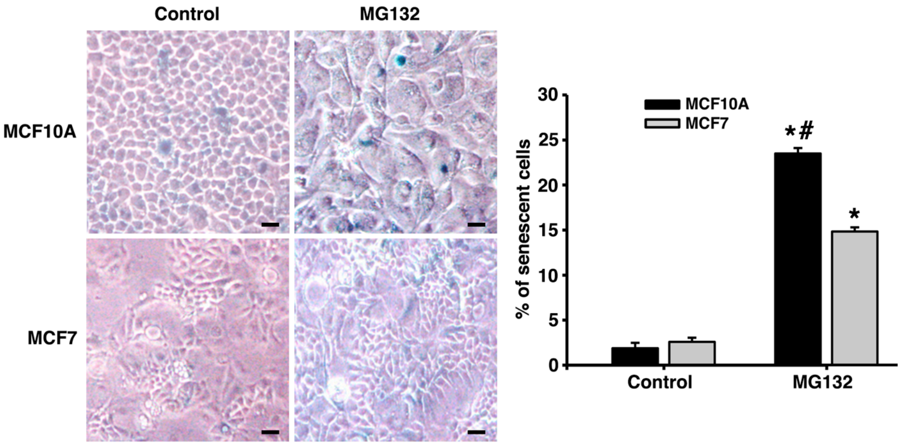

Supplementary Figure S2. Analysis of cellular senescence in MCF7 and MCF10A induced by proteasome inhibition. Representative images of β-galactosidase staining and proportion of senescent cells in both cell types. Scale bar 30 μm.

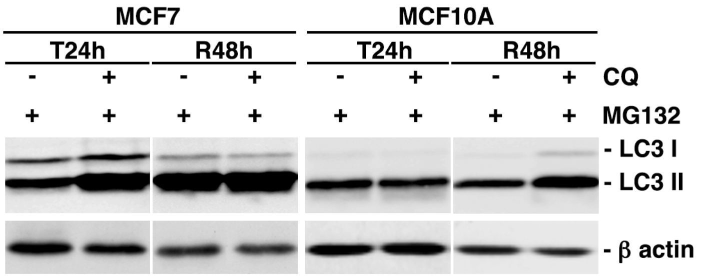

Supplementary Figure S3. Analysis of autophagy flux in MCF7 and MCF10A cells following 24 hours of 1 μM MG132 treatment in the absence and in the presence of 5 μM of chloroquine (CQ) (T24h), and 48 hours after elimination of MG132 (R48h). Chloroquine was added 5 hours before cells were collected. Note that autophagy flux was higher in MCF7 cells after 24 hours of MG132 treatment but in MCF10A cells this increase was observed during the recovery phase.
